# Organelle Genomes and Transcriptomes of *Nymphaea* Reveal the Interplay between Intron Splicing and RNA Editing

**DOI:** 10.3390/ijms22189842

**Published:** 2021-09-11

**Authors:** Zheng-Shan He, Andan Zhu, Jun-Bo Yang, Weishu Fan, De-Zhu Li

**Affiliations:** 1 Germplasm Bank of Wild Species, Kunming Institute of Botany, Chinese Academy of Sciences, Kunming 650201, China; hezhengshan@mail.kib.ac.cn (Z.-S.H.); zhuandan@mail.kib.ac.cn (A.Z.); jbyang@mail.kib.ac.cn (J.-B.Y.); 2 University of Chinese Academy of Sciences, Beijing 100049, China

**Keywords:** *Nymphaea*, organelle genomes, transcriptomes, intron splicing, RNA editing

## Abstract

Posttranscriptional modifications, including intron splicing and RNA editing, are common processes during regulation of gene expression in plant organelle genomes. However, the intermediate products of intron-splicing, and the interplay between intron-splicing and RNA-editing were not well studied. Most organelle transcriptome analyses were based on the Illumina short reads which were unable to capture the full spectrum of transcript intermediates within an organelle. To fully investigate the intermediates during intron splicing and the underlying relationships with RNA editing, we used PacBio DNA-seq and Iso-seq, together with Illumina short reads genome and transcriptome sequencing data to assemble the chloroplast and mitochondrial genomes of *Nymphaea* ‘Joey Tomocik’ and analyze their posttranscriptional features. With the direct evidence from Iso-seq, multiple intermediates partially or fully intron-spliced were observed, and we also found that both *cis*- and *trans*-splicing introns were spliced randomly. Moreover, by using rRNA-depleted and non-Oligo(dT)-enrichment strand-specific RNA-seq data and combining direct SNP-calling and transcript-mapping methods, we identified 98 and 865 RNA-editing sites in the plastome and mitogenome of *N.* ‘Joey Tomocik’, respectively. The target codon preference, the tendency of increasing protein hydrophobicity, and the bias distribution of editing sites are similar in both organelles, suggesting their common evolutionary origin and shared editing machinery. The distribution of RNA editing sites also implies that the RNA editing sites in the intron and exon regions may splice synchronously, except those exonic sites adjacent to intron which could only be edited after being intron-spliced. Our study provides solid evidence for the multiple intermediates co-existing during intron-splicing and their interplay with RNA editing in organelle genomes of a basal angiosperm.

## 1. Introduction

The gene expression of organelle genomes (chloroplast and mitochondrial genomes) in land plants involves complex post-transcriptional processing steps, such as intron splicing and RNA editing, etc. [1,2,3]. Most of the organelle introns in higher plants belong to a group II intron that generally consists of an RNA and a protein component, but is in a much-degenerated form [4]. The RNA component is a ribozyme which has six stem-loop domains (D1 to D6) and is incapable of self-splicing in contrast to bacterial group II intron. The protein component is an intron-encoded protein (IEP) located in domain IV of the RNA component. The IEP is comprised of reverse transcriptase (RT) domain, domain X (thumb domain of RT), DNA binding domain (D) and endonuclease domain (En) [4,5]. Group II introns can be divided into three families according to exon recognition mechanism: II-A (generally in mitochondria), II-B (generally in plastid) and II-C (plastid-like in bacteria) [6,7]. Chloroplast genome (ptDNA) and mitochondrial genome (mtDNA) of vascular plants each encode about 20 group II introns [7,8]. Some introns are fragmented and must assemble in *trans* to form a splicing-competent structure. *Trans*-spliced group II intron arises from DNA recombination events that interrupt an initially unbroken *cis*-spliced group II [2]. Angiosperm mtDNA usually contains five to six *trans*-spliced introns with fragmentation site in domain IV, mostly spreading in genes *nad1* (*nad1*-i1, *nad1*-i3, some of the *nad1*-i4), *nad2* (*nad2*-i2), *nad5* (*nad5*-i2, *nad5*-i3), and one case in *cox2* [2,9]. In contrast, in ptDNA, only *rps12* intron 1 (*rps12*-i1) is *trans*-spliced which is disrupted in domain III [10].

RNA editing, a co/post-transcriptional alteration of RNA transcripts, is prevalent within organelles [1,11]. RNA editing in plants was first identified as deamination of cytidine to uridine (C-to-U) in mitochondria [12,13,14,15], and then in chloroplast of maize [16]. C-to-U RNA editing exists in all land plants lineages except Marchantiid liverworts [17] and plastome of *Welwitschia* [18], but is absent from algae and prokaryotes. RNA editing is thus considered to be evolved after the terrestrialization of green plants about 500 million years ago [1]. Reverse U-to-C RNA editing has been nearly lost in angiosperms and gymnosperms [1]. There are 20–60 and 300–600 RNA editing sites in plastids and mitochondria of most angiosperms, respectively [19]. A decrease in organellar RNA editing sites along the angiosperm evolution is documented widely [20,21,22,23,24,25]. Most of the RNA editing sites are located at protein-coding regions and a few sites have been identified in intronic and untranslated regions (UTR) [26,27]. In addition, to ensure the production of functional proteins, C-to-U editing can also affect the RNA secondary structure, which is crucial to RNA stability and intron splicing [1].

Nymphaeales is the sister clade to all other extant angiosperms except Amborellales [28]. There are 184 and 824 RNA-editing sites in the plastome and mitogenome of *Amborella trichopoda* (Amborellales), respectively [23,24], and 781 sites in the mitochondrial genome of *Liriodendron tulipifera* (Magnoliales) [29]. So far, the genome-wide experimental evidence of intron splicing and RNA editing information is lacking in Nymphaeales. In this study, we assembled the whole plastid and mitochondrial genome of *Nymphaea* ‘Joey Tomocik’ (Nymphaeales) with PacBio long reads and Illumina short reads which were generated in an ongoing *Nymphaea* ‘Joey Tomocik’ genome project. By utilizing the high-depth Iso-seq reads and RNA-seq reads, and additionally rRNA-free strand-specific RNA-seq data from the same individual, we investigated the full RNA landscape of plastid and mitochondria of *Nymphaea*, including its intron splicing and RNA editing. We further discussed the interplay of intron splicing and RNA editing.

## 2. Results and Discussion

### 2.1. Complete Organelle Genomes of N. ‘Joey Tomocik’

We used both PacBio and Illumina DNA-seq reads to assemble the organelle genomes and cross-checked the assemblies to obtain the solid complete genomes. The PacBio sequencing generated 3,239,192 (19.68 Gb) single-molecule long subreads in total, with an average length of 6077 bp, and N50 of 9200 bp. The Illumina DNA-seq generated 418,782,912 (73.92 Gb) raw paired-end short reads and 60.72 Gb clean reads were kept for the following assembly after quality control (Supplementary Table S1).

Chloroplast genome of *N.* ‘Joey Tomocik’ was successfully assembled into one circular molecular (total length 159,960 bp) by Organelle_PBA using PacBio reads. GetOrganelle failed to assemble the plastome. NOVOPlasty also successfully assembled the circular chloroplast genome (total length of 159,968 bp) with an average coverage of 13,459 (Supplementary Figure S1). These two assemblies were nearly identical except for four SNPs and 14 mononucleotide indels. Considering the error-prone nature of PacBio reads, the assembly by NOVOPlasty (159,968 bp) was chosen as the final chloroplast genome of *N.* ‘Joey Tomocik’.

**Figure 1:**
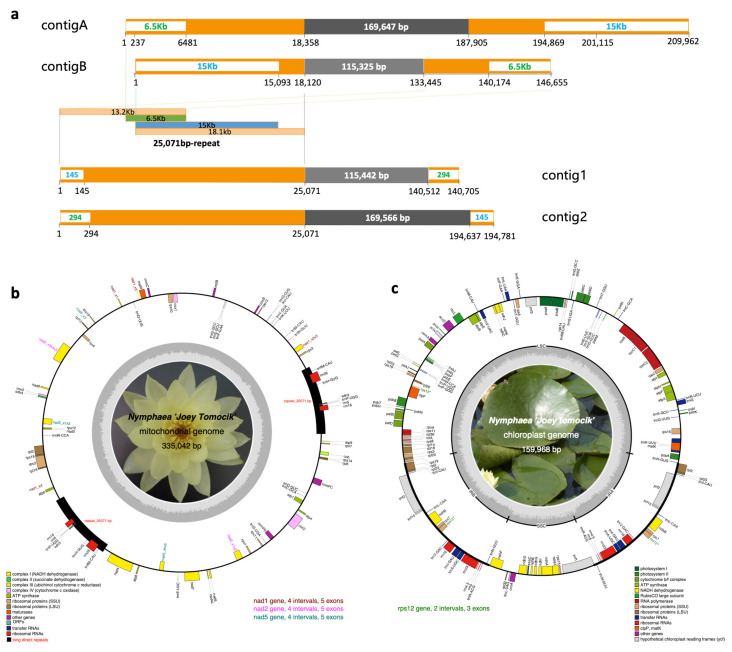
Assembly and annotation of *N.* ‘Joey Tomocik’ organelle genomes. (**a**) Mitochondrial genome contigs assembled either from PacBio long reads (contigA and contigB) or from Illumina short reads (contig1 and contig2). Orange boxes indicate repeat regions (25,071 bp in total length), and the blue and green texts with white backgrounds in orange boxes are overlapped regions to concatenate two contigs end to end. The components of the 25071bp-repeat are indicated in light orange, green and blue boxes. Grey boxes are unique regions in each contig. (**b**) Complete mitochondrial genome of *N.* ‘Joey Tomocik’. (**c**) Complete chloroplast genome. Gene annotations are color-coded in different functional groups, and the grey inner circles indicate the GC content. The four *trans*-splicing intron-containing genes (*nad1, nad2, nad5* in mitogenome, *rps12* in chloroplast genome) are highlighted. The 25,071 bp long direct repeat is indicated by black box.

Plant mitogenomes are fluid in genome structure via frequent genomic rearrangements [30]. We took the advantage of PacBio long reads to assemble this complicated genome. Initially, Organelle_PBA only generate one contig of 194,633 bp assembled from 48,796 mapped reads. Utilizing the iterative approach (10 rounds) for mitogenome assembling, 88,554 (2.73%) single-molecule long reads with a total length of 643,311,976 bp (3.27%) were recruited to produce five contigs with a total length of 580,548 bp. Based on a BLASTN search against the mitochondrial genome of *Nymphaea colorata* (NC_037468), two contigs were selected as of mitochondrial assembly (contigA and contigB, Figure 1a). contigA of 209,962 bp was assembled from 805 reads, and contigB of 146,655 bp assembled from 539 reads. These two contigs can be merged into a circular sequence by overlapping 15 kb and 6.5 kb end to end. The resulting consensus circular mtDNA is 335,079 bp in length. After three rounds of polish with Arrow and further five rounds of polish with Pilon, quadripartite mitogenome of 335,137 bp was assembled. To cross-check our high-quality assembly by PacBio long reads, Illumina short reads were also employed to assemble the mitogenome by NOVOPlasty (Supplementary Figure S1). The two longest contigs (contig1 and contig2, Figure 1a) with a length of 140,705 bp and 194,781 bp were chosen as candidates. These two contigs shared the same 25,071 bp at the terminal. By comparing to the PacBio assembly, we connected them by overlapping 145 bp and 294 bp at both ends to get a circular sequence of 335,042 bp which showed exactly collinearity to the PacBio assembly. The final mitochondrial genome of *N.* ‘Joey Tomocik’ was 335,042 bp (Figure 1b).

To evaluate our mitochondrial genome assembly procedures, we downloaded the raw PacBio RSII reads of *N. colorata* from BIG Data Center (http://bigd.big.ac.cn, accessed on 19 March 2021) under accession number CRR058166 and assembled the mitogenome using the same pipeline as we did for the *N.* ‘Joey Tomocik’ mitochondrial genome assembly. The assembly of *N. colorata* from our pipeline showed similar length and perfect collinearity with the published individual (NC_037468) (Supplementary Figure S2). This result suggested that the mitochondrial genome of *N.* ‘Joey Tomocik’ and that of *N. colorata* (MW644617 and NC_037468) vary substantially in their genome structures. Mitogenomes often contain highly variable intergenic regions of endogenous or sequences from foreign origins and undergo frequent recombination events aroused by repeat sequences. The 25,071 bp repeat in *N.* ‘Joey Tomocik’ was not shown in the mitogenome of *N. colorata* [31], further supporting that mitogenome configurations could be different between closely-related species [30].

Chloroplast genome of *N.* ‘Joey Tomocik’, exhibiting typical circular quadripartite structure, is comprised of a large single copy (LSC, 90,025 bp) region, a small single copy (SSC, 19,533 bp) region and a pair of inverted repeats (IRs, 25,205 bp). We found 114 unique genes (17 duplicated genes in IR) in the chloroplast genome, including 4 rRNA, 30 tRNA, and 80 protein-coding genes (Figure 1c), which is the same with gene content in that of *Nymphaea mexicana* and *N. colorata*. GC contents of whole genome, LSC, SSC, and IR are 39.1%, 37.8%, 34.2%, and 43.3%, respectively. Comparative genomics revealed that plastome of *N.* ‘Joey Tomocik’ show identical gene content and gene order compared to other available basal angiosperms (Table 1).

Mitogenome of *N.* ‘Joey Tomocik’ has 65 unique genes, including 3 rRNAs, 21 tRNAs, and 41 protein-coding genes (Table 1, Figure 1b), which represents nearly all the functionally diverse repertoire of protein-coding genes in land plants except for two genes (*rpl6* and *rps8*) absent in all seed plants [32]. Compared to other available mitogenomes of basal angiosperms, including those of *Amborella*, *Nymphaea*, *Schisandra* (Austrobaileyales), and *Liriodendron*, mitogenome of *N.* ‘Joey Tomocik’ has the most complete gene sets (Table 1). The total length of insertions from plastid origins was about 20.8 kb (6.2%) (Supplementary Table S2).

We double-checked the annotations of organelle genomes by comparing them with the well-annotated species (thale cress, rice, wheat, tobacco, and *Amborella*) in GenBank database, and by comparing with the mapped Iso-seq reads. We found that the boundaries between *nad4* exon 2 and exon 3 in most species were misannotated. By using multiple sequence alignments, together with the mapped Iso-seq transcripts, we found that the end of *nad4* exon 2 should be AGTCGG (elongate two nucleotides GG) and the start of *nad4* exon 3 should be AACATA (eliminate two nucleotides CG). The amino acid should be corrected to arginine (R) instead of proline (P) (Supplementary Figure S3). Other corrections with the help of Iso-seq transcripts mapping results are exon 2 of *rpl16*, exon 1, and exon 2 of *cox2*. Full-length transcripts, as empirical evidence, could greatly improve the annotation accuracy of organelle genomes by automatic pipelines.

### 2.2. Co-Transcribed Genes in Organelle Genomes of N. ‘Joey Tomocik’

The Iso-seq data provides valuable resources to detect the co-transcribed genes in both chloroplast and mitochondrial genomes. Of all the 441,656 corrected full-length Iso-seq transcripts, 3121 and 4190 transcripts were mapped to chloroplast and mitochondrial genome by GMAP, respectively. After manual refinement according to the full covering of coding regions, 956 and 337 Iso-seq transcripts were kept for the co-transcription detection. We identified 21 and five polycistronic transcriptional units (PTUs) in plastome and mitogenome, respectively. After concatenated adjacent overlapping cistronic transcripts into the same PTU, we finally got 18 and five PTUs in plastome and mitogenome, respectively (Table 2). The five mitochondrial PTUs covered 11 protein-coding genes, and the gene cluster *rpl2-rps19-rps3* is found in many mitogenomes of seed plants [29], indicating the retaining of a conserved co-transcribed gene cluster along with evolution. Among the 18 plastid PTUs, the largest ribosomal protein operon (PTU13, *trnI-CAU, rpl23, rpl2, rps19, rpl22, rps3, rpl16, rpl14, rps8, infA, rpl36, rps11, rpoA*) contains 13 genes, followed by RNA polymerase and ATP synthase operon (*trnE-UUC, trnY-GUA, trnD-GUC, psbM, rpoB, rpoC1, rpoC2, rps2, atpI, atpH, atpF, atpA*) with 12 genes, and NADH dehydrogenase operon (PTU18, *ycf1*(partial), *rps15, ndhH, ndhA, ndhI, ndhG, ndhE, psaC, ndHD*) with nine genes. In total, 77 (presenting 96.25%) chloroplast protein-coding genes are co-transcribed with other genes, providing more evidence for the wide existence of co-transcription in plastomes. We also found a strange PTU covered *clpP*, *rps12*exon 1, *rps12*exon 2, *rps12*-i2 by Iso-seq transcript “c260475/f1p0/1584”. This poly-cistron is the consequence of *trans*-splicing of *rps12*-i1 and thus was not included here.

We expanded several operons (genes covered by one PTU) comparing to 20 operons in barley chloroplast [33]. For example, PTU3 in plastome of *Nymphaea* contains three operons and two monocistronic transcripts (*trnD-GUC*, *psbM*) in barley. The operon which contains four rRNA genes in barley was not found here.

### 2.3. Diverse Intron Splicing Intermediates in Organelle Genome

**Figure 2:**
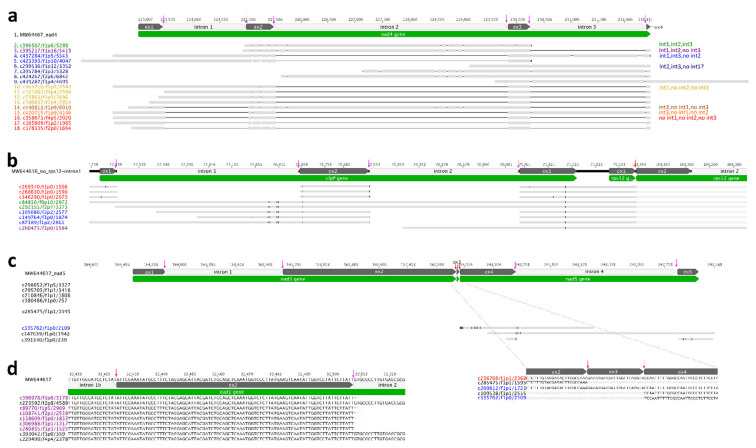
Evidence of intron splicing in *Nymphaea*. (**a**) *cis*-splicing of *nad4* intron 1, intron 2, and intron 3 in mitogenome. Different colors indicate different splicing intermediates (all eight observed are listed in the right panel). Spliced introns are indicated as black lines, and “ex” is the abbreviation for exon. All *cis*-splicing sites are indicated by pink arrows. (**b**) *cis*-splicing of *clpP* intron 1 and intron 2 and *trans*-splicing of downstream *rps12* gene in plastome. Transcripts in red are the product of all two introns of *clpP* spliced; Transcripts in green are intact *clpP* gene with both two introns unspliced; Transcripts in blue are products of only intron 2 of *clpP* spliced; Transcripts in purple is the product of *trans*-spliced of *rps12* intron 1; *Trans*-splicing site is indicated by red arrow. (**c**) *trans*-splicing of *nad5* gene in mitogenome. Transcript in red is the product of all five introns spliced; transcript in blue is the product of intron 2 and intron 3 spliced and *cis*-splicing intron 1 unspliced. (**d**) *cis*-splicing intron is spliced earlier than *trans*-splicing intron (in *nad1* gene).

*N.* ‘Joey Tomocik’ chloroplast genome contains one *trans*-splicing intron (*rps12*-i1) and its mitogenome contains six (*nad1*-i1, *nad1*-i3, *nad1*-i4, *nad2*-i2, *nad5*-i2, *nad5*-i3) *trans*-splicing introns. The other identified introns are *cis*-spliced. 25 splicing events (53.33%) detected by Iso-seq transcripts. Another 14 splicing events could be detected by Trinity transcripts (86.67%) (Table 3). With the help of full-length Iso-seq transcripts, intermediates of *cis*-spliced intron were detected. For example, all eight possible products of intron-spliced were identified in the mitochondrial *nad4* gene, including the transcript that showed intron 1 and intron 3 was spliced, but intron 2 was un-spliced (Figure 2a). A similar phenomenon can be found in chloroplast genome, too. For example, three different products were identified in *clpP* gene except intron 1 spliced but intron 2 un-spliced (Figure 2b). These cases provide strong evidence that the intron splicing does not follow a particular order but can be randomly split into any possible intermediates and co-exists in a cell. Although this hypothesis has been documented in the *trans*-splicing *nad5* gene of maize mitogenome, they did not detect all the possible intermediates of intron-splicing [34]. Our observations provide unambiguous support that all possible intermediates could simultaneously exist in the cell.

Additionally, we also found the dynamic configurations between *cis*- and *trans*-splicing. From Iso-seq transcripts mapping results, four *trans*-splicing events were detected (*rps12*-i1, *nad1*-i3, *nad5*-i2, *nad5*-i3) and the other three *trans*-splicing events (*nad1*-i1, *nad1*-i4 and *nad2*-i2) were further detected by Trinity transcripts (Table 3, Figure 2, Supplementary Figure S4). Our PTU analysis revealed that *rps12*-5′ (exon 1 and intron 1a) could be co-transcribed with upstream *clpP* gene and downstream *rpl20* gene, and *rps12*-3′ (intron 1b, exon 2-intron 2, exon 3) could be co-transcribed with downstream *rps7* and *ndhB* gene (PTU11 and PTU15 in Table 2). However, the transcript (c260475/f1p0/1584) showed that the *trans*-splicing event occurred, but the *cis*-splicing intron remained un-spliced (Figure 2b). It is possible that *trans*-splicing happened before *cis*-splicing, which was also observed in mitochondria *nad5*. We found the transcript (c335762/f1p0/2109) which showed *nad5*-i3 *trans*-spliced and *cis*-splicing *nad5*-i4 un-spliced. Likewise, the transcript c209612/f1p1/1721 displayed *nad5*-i2 and *nad5*-i3 spliced (*trans*-splicing) while *nad5*-i1 (*cis*-splicing) was un-spliced (Figure 2c). The opposite case was also detected, referring to the *cis*-splicing *nad1*-i2 spliced earlier than *trans*-splicing *nad1*-i1b (Figure 2d). We checked all genes containing both *cis*-splicing and *trans*-splicing introns using Trinity transcripts mapping results and we found *cis*-splicing intron spliced before *trans*-splicing intron was also prevalent. Taken together, we proposed that both *cis*- and *trans*-splicing intron spliced randomly, generating diverse intermediates.

### 2.4. RNA-Editing in Organelle Genomes of Nymphaea

**Figure 3:**
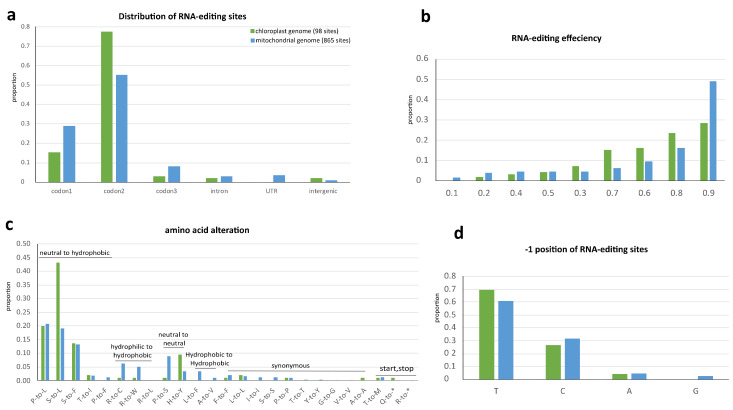
Comparison of RNA-editing in plastome and mitogenome of *N.* ‘Joey Tomocik’. The hydropathic character of amino acid was assigned according to IMGT class [37]. (**a**) Distribution of RNA-editing sites; (**b**) RNA-editing efficiency at each editing site; (**c**) Amino acid alteration of RNA-editing; (**d**) Nucleotide preference at the -1 position of editing site.

Since plant organellar transcripts generally do not have poly-A tail and posttranscriptional polyadenylation accelerating their degradation [35], we use strand-specific RNA-seq (ssRNA-seq) to get the full landscape of organellar RNA editing. With the strict filter procedures as described in methods, we eventually identified 98 and 865 RNA editing sites in chloroplast and mitochondrial genome, respectively (Figure 3a, Supplementary Tables S3 and S4). We identified an RNA-editing site in *rps12*-i1 (*trans*-splicing intron) which was not reported before (Supplementary Figure S5), but we did not detect the previously reported two sites in *rps12*-i2 [36]. Additionally, no editing site was observed in tRNA or rRNA genes. The C-to-U site found in *trnM-CAU* of *Amborella* was absent in *N.* ‘Joey Tomocik’ [24]. These probably due to the strict filter processing in our identification pipeline.

116 plastid and 385 mitochondria RNA-editing sites were identified by mapping Iso-seq transcripts to the corresponding genomes, in which 62 and 50 sites were absent from the final RNA-editing dataset, respectively. Most of these excluded sites are not shown in the SNPs called by bcftools, and all are lacking Trinity transcripts support (Supplementary Table S5). The insufficient coverage of Iso-seq transcripts against mitogenome led to the low number of identified RNA editing sites. We also use REPACT3 to predict the editing sites to evaluate how it was consistent with the experimental result. Some 76 and 615 predicted sites were present in the experimentally identified RNA-editing sites of plastome and mitogenome, respectively (Supplementary Tables S3 and S4), covering more than 70% of the total sites.

Most of the editing sites fall into coding regions (Figure 3a). The total 94 sites in plastome coding regions are distributed into 36 genes and 807 mitogenome coding region editing sites spread in all its 41 genes (Supplementary Tables S6 and S7). Among these sites, 89 and 734 sites in plastid and mitochondria are non-synonymous RNA-editing (Supplementary Tables S3 and S4). There is one-stop codon (in *petD*) in plastid and two-stop codons (in *atp6* and *ccmFC*) in mitochondria generated by RNA-editing with more than 86% editing efficiency, resulting in truncation of conserved protein sequences (Supplementary Tables S3 and S4). In both organelle genomes, RNA-editing machinery preferentially targets the second and first base of codon (Figure 3a), consequently increase hydrophobicity of all the amino acids after 12 non-synonymous changes according to the Kyte–Doolittle scale and IMGT class (I, V, L, F, C, M, A, W, G, T, S, Y, P, H, N, D, Q, E, K, R) [37,38] (Figure 3c).

The RNA-editing efficiency (VAF) of all sites was calculated. More than 80% of sites in both organelle genomes were efficiently edited (>0.6). The average editing efficiencies of CDSs, introns, UTRs, and intergenic regions of the mitogenome are 0.802, 0.796, 0.693, and 0.625, respectively. The average editing efficiencies of CDSs, introns (only two sites), and intergenic regions (only two sites) of plastome are 0.761, 0.772, and 0.278, respectively (Figure 3b).

The nucleotide 5′-adjacent to the edited C (−1 position) is often a pyrimidine in *Arabidopsis* mitogenome [39] and in *Amborella* plastome [24]. This is in common in both organelle genomes of *Nymphaea*, pyrimidines dominating more the 93% at the −1 position (Figure 3d). Overall, the RNA editing target codon preference, the tendency of increasing protein hydrophobicity and the sites’ distributions displayed a similar tendency in both organelles. It may be concluded that RNA editing in both organelle genomes shared a common evolutionary origin and mechanism, as implied by an early study [40].

To compare the available RNA-editing events in basal angiosperms, we obtained the plastome RNA-editing information of *L. tulipifera* and *A. trichopoda* by downloading the available SRR data (Supplementary Table S8) and conducted the same identification procedures as used in our study (see Section 3.7). Some 89 and 169 RNA-editing sites were identified in chloroplast genomes of *L. tulipifera* and *A. trichopoda*, respectively (Supplementary Tables S9 and S10). Some 827 RNA-editing sites of *L. tulipifera* mitogenome were retrieved from the modified genbank file of KC821969 by PREPACT3 webserver.

**Figure 4:**
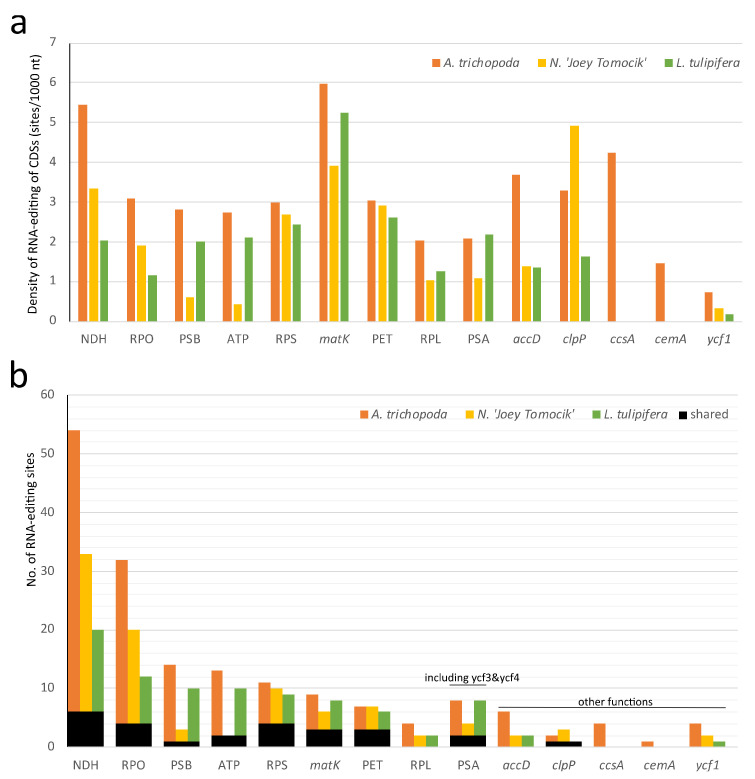
Distribution of RNA-editing sites in 10 groups of plastomes of three basal angiosperms. (**a**) RNA-editing density per 1000 nucleotides in each protein-coding gene (or group); (**b**) Number of RNA-editing sites in each CDS (or group). NDH (NADH dehydrogenase, *ndhA, ndhB, ndhC, ndhD, ndhE, ndhF, ndhG, ndhH, ndhJ,* and *ndhK),* RPO (RNA polymerase, rpoA, *rpoB, rpoC1,* and *rpoC2*), PSB (Photosystem II, *psbA, psbB, psbD, psbE, psbF, psbH, psbJ, psbK, psbL, psbN,* and *psbZ*), ATP (ATP synthase, *atpA, atpB, atpE, atpF,* and *atpI*), RPS (Ribosomal protein small, *rps2, rps16, rps14, rps3, rps8, rps18, rps4,* and *rps7*), PET (Cytochrome b/f complex, *petA, petB, petD, petG,* and *petL*), RPL (Ribosomal protein large, *rpl14, rpl16, rpl20, rpl22, rpl23,* and *rpl32*), PSA (Photosystem I, *psaA, psaC, psaI, ycf3,* and *ycf4*). The orange, yellow, and green bars represent species *A. trichopoda*, *N.* ‘Joey Tomocik’, and *L. tulipifera*, respectively; the black bar shows the shared sites among the three species.

In the plastid genome, 48, 36, and 41 protein-coding genes of *Amborella*, *Nymphaea,* and *Liriodendron* showed RNA editing, respectively. In all three species, NDH, *matK,* and *clpP* have the highest density of RNA-editing sites. For individual genes, *ndhD, rpoC1, matK, rpoB,* and *ndhB* own the highest number of editing sites (Figure 4, Supplementary Table S6).

**Figure 5:**
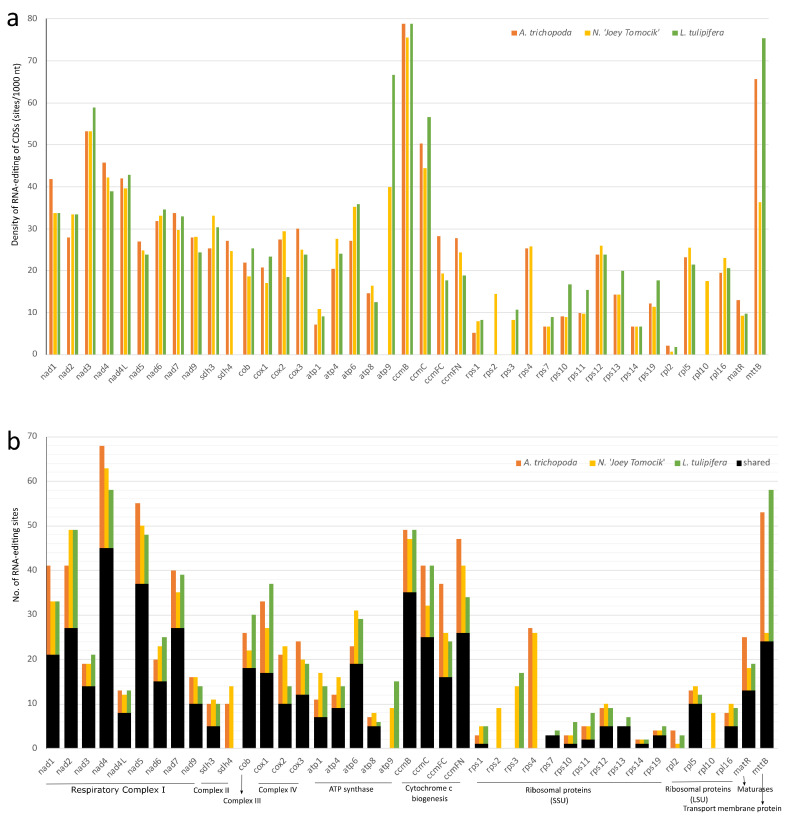
Distribution of RNA-editing sites in 41 protein-coding genes of mitogeomes of three basal angiosperms. (**a**) RNA-editing density per 1000 nucleotides in each CDS; (**b**) Number of RNA-editing sites in each CDS. The orange, yellow, and green bars represent species *A. trichopoda*, *N.* ‘Joey Tomocik’, and *L. tulipifera*, respectively; the black bar shows the shared sites among the three species.

In mitochondria, all 41 protein-coding genes of *Nymphaea* are RNA-edited. *rpl10* and *rps2* gene of *A. trichopoda* and *L. tulipifera* both have no RNA-editing sites (Supplementary Table S7). In all three species, *ccmB, mttB, nad3, ccmC, nad4,* and *nad4L* have the highest density of RNA-editing sites, and *nad4*, *nad5*, *ccmB*, *nad2*, *mttB*, and *ccmFN* have the highest total number of RNA-editing sites (Figure 5, Supplementary Table S7).

The high level of C-to-U RNA-editing identified by empirical data in all three basal angiosperms, and high proportion of shared sites among the three representatives especially in mitogenomes concluded that the ancestral angiosperm possessed a relatively high level of RNA-editing in organelle genomes, with extensive loss in different lineages. To test whether RNA-editing sites have phylogenetic signals, we selected the representative CDSs (*nad4* of mitogenome, *ndhD*, *rpoC1,* and *matK* of plastome) and only aligned those edited sites (Supplementary Figure S6). There was no obvious phylogenetic signal except the total number variation. Each species had exclusive editing sites which did not share with the other two species, implying that RNA-editing sites undergo independent lost after species divergence.

### 2.5. Interplay between RNA-Editing and Intron-Splicing

**Figure 6:**
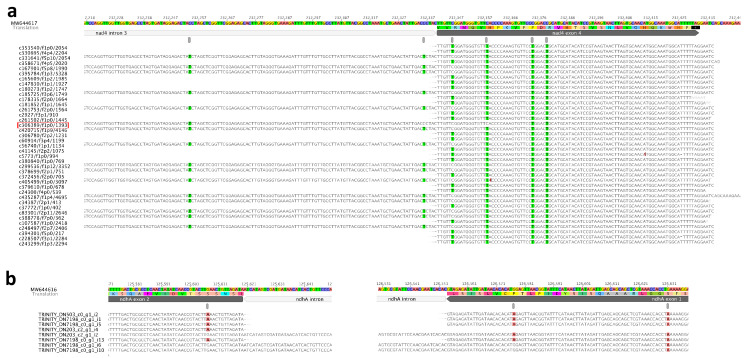
The interplay between intron-splicing and RNA-editing in *Nymphaea*. (**a**) *nad4* gene of mitogenome; (**b**) *ndhA* gene of plastome. Highlighted based are RNA-editing sites.

A previous study demonstrated that some exonic RNA-editing sites near intron junctions remain unedited in pre-RNAs, while more distal sites could be edited more efficiently [1,41,42,43]. Our result shows that the RNA-editing site in mitochondrial *nad4* exon 4 which is 6 bp downstream intron 3, cannot be edited if intron 3 was un-spliced. While the following three sites which are 18 bp, 34 bp, and 39 bp downstream intron 3 have not been affected (Figure 6a). The VAFs of these four sites are 0.639, 0.935, 0.719, and 0.776 respectively. The Iso-seq transcript “c306389/f1p0/1393” denoted the three sites (one in *nad4*-i3, two in exon 4) near the intron-exon boundary that are not edited. Therefore, it is possible that editing sites near the intron-exon junction in un-spliced pre-mRNA are processed late (Figure 6a). The same phenomenon was also observed in two editing sites in plastid *ndhA* gene, the site 24 bp upstream intron-exon junction, and the site 13 bp downstream intron-exon junction (Figure 6b). The other seven spliced-edited events (intron-spliced then exonic RNA-edited) were also detected in seven exons adjacent to introns (Supplementary Figure S7). Exonic affected RNA-editing sites exhibited a similar extent and pattern, suggesting that they might be recognized and edited by the same RNA-editing factor. It further indicated that the recognition target of some genes could only be formed after intron splicing.

We also investigated the interplay between *trans*-splicing intron and the nearby editing sites. The spliced-edited event was only detected in mitochondrial *nad1*-i3a and *nad1* exon 3 by Trinity transcripts. However, all RNA-editing sites in *nad1* exon 3 were fully edited in the Iso-seq transcripts mapping result (Supplementary Figure S8a,b). While in *nad4* exon 4 downstream *nad4*-i3, the spliced-edited events were both observed in the Iso-seq and Trinity transcripts mapping results (Figure 4a, Supplementary Figure S8c). Based on these results, we speculated that those *trans*-spliced introns should be assembled into a complete intron before the splicing process started.

We examined the distance between splicing boundaries and the nearest editing sites to illustrate whether it was an influencing factor. The longest distance from partially edited sites to intron boundary was 39 bp (*nad7* exon 3), and the shortest was 2 bp (*nad4* exon 2) (Supplementary Figure S7). Besides un-detected RNA-editing sites in exon near intron, there are many exonic sites that were not affected by intron-splicing (Supplementary Figure S9).

RNA editing can occur before intron splicing in pre-mRNAs. Both *nad1* intron and exon had editing sites observed in un-spliced cDNA of mitogenome of *Petunia* [44]. Analogous data were also reported, as for *rps10* exon and unspliced intron in mitogenome of potato [45], *nad2* mRNAs in *Oenothera* mitochondria [46]. The RNA-editing processes in intron and exon probably happened at the same time, as observed in our study (Figure 2, Figure 6 and Figure S8).

RNA editing is crucial for intron splicing by improving the base pairing of stem-loop secondary structure impaired by DNA mutation. The sites in domain VI and V of *nad4*-i3 which are 5 bp and 87 bp upstream exon 4 (VAF are 0.867 and 0.896 each) are vital for intron-splicing, as well as two sites in domain V of *nad7*-i4 (Supplementary Figure S10). Two editing sites in domain VI of *nad1*-i1 [47] and one in *nad1*-i3 had been found critical for intron-splicing in *Oenothera* [48]. Likewise, one editing site in domain VI of *nad1*-i4 in wheat [49]. The sites in the stem of domain I and IV of *nad2*-i2 in *Oenothera* [46] and wheat [50] are also indispensable, as well as editing site in domain VI of *nad5*-i2 in wheat [49], in domain V of *nad7*-i3 and *nad7*-i4 in maize [51].

## 3. Materials and Methods

### 3.1. Genomic DNA Isolation and Sequencing

*Nymphaea* ‘Joey Tomocik’ is planted at Kunming Botanic Garden, Kunming Institute of Botany, Chinese Academy of Sciences, Kunming, Yunnan, China. Fresh petals and stamens from budding flowers were collected and quickly frozen in liquid nitrogen for further DNA and RNA extraction. Total DNA was extracted using a modified cetyl-trimethyl-ammonium-bromide (CTAB) protocol [52], and used for genome sequencing. One PacBio library with insert size of 20 kb was constructed using SMRTbell Template Prep Kits 1.0, and subsequently five SMRT cells were sequenced by PacBio Sequel platform using Sequel^TM^ sequencing Kit 1.2.1 at Wuhan Institute of Biotechnology (Wuhan, China). The same batch of extracted genomic DNA was used to construct Illumina libraries. One library with insert size of 400 bp was made using Illumina TruSeq Nano DNA Library Prep Kits and sequenced with 2 × 150 bp paired-ends (PE) by Illumina Hiseq X Ten at Nextomics Biosciences Co., Ltd. (Wuhan, China), generating about 60 Gb clean data (from the *N.* ‘Joey Tomocik’ genome project).

### 3.2. Organelle Genome Assembly and Annotation

Complete chloroplast and mitochondrial genomes of *N.* ‘Joey Tomocik’ were both assembled from a combination of two genomic sequencing datasets, which was the PacBio single-molecular long reads from all five SMRTcells and the Illumina Hiseq clean data of approximate 60 Gb.

Organelle_PBA [53] was used to assemble the organelle genomes from all PacBio long reads, using the plastome of *Nymphaea mexicana* (NC_024542) [54] and the mitogenome of *Nymphaea colorata* (NC_037468) [31] as the reference genomes. An iterative approach introduced by Kovar et al. [55] was also applied to assemble the mitogenome. Specifically, we firstly mapped raw long reads to a reference genome of closely related species, *N. colorata* (NC_037468) to extract a subset of raw reads that likely belong to the mitochondrial genome of interest. Then we utilized canu v2.1 [56] to de novo assemble these extracted raw reads. The process was repeated for 10 rounds and using the new draft genome derived from the prior round as the subsequent reference to recruit additional reads. Each round of assembly ends with the de novo reconstruction of the draft genome from recruited reads. With this approach, we obtained two scaffolds and then manually assembled to a circular complete mitogenome by inferring the repeat regions. All draft assemblies were then polished by Arrow [57] and/or Pilon [58].

To verify the assemblies from the PacBio long reads and obtain more accurate organellar genomes, we reassembled the organellar genomes from the Illumina NGS reads by GetOrganelle v1.7.1 [59] and NOVOPlasty v4.2 [60]. For GetOrganelle, ‘get_organelle_from_reads.py’ with default parameters “-R 15-k 21,45,65,85,105” was executed. For NOVOPlasty, in “chloro” mode, the default RuBP sequence was chosen as seed sequence, and the plastome of *N. mexicana* (NC_024542) was used as the reference sequence. In “mito_plant” mode, four CDS sequences (*atp1*, *atp6*, *cox1*, *matR*) from the mitogenome of *Arabidopsis thaliana* (NC_037304) were selected as seed sequence and the same plastome (NC_024542) was set as “Chloroplast sequence,” leaving the “Reference sequence” empty. The assembled mitochondrial scaffolds were also manually joined together guided by PacBio assembly to generate the complete mitogenome.

Chloroplast genome was annotated by PGA [61] using plastome of *N. Mexicana* (NC_024542) and *A. thaliana* (NC_000932) as references. Mitochondrial genome was annotated by Geseq [62] using mitogenome of *N. colorata* (NC_037468) and *A. thaliana* (NC_037304) as references. The missing annotations, exact gene, and intron boundaries were manually checked and edited in Geneious R9.1.8 (Biomatters Ltd., Auckland, New Zealand). The assembled and annotated chloroplast and mitochondrial genomes of *N.* ‘Joey Tomocik’ were submitted to the GenBank (assigned accessions MW644616 and MW644617, respectively). Fully annotated organelle genome diagrams were drawn by OrganellarGenomeDRAW (OGDRAW) [63].

Repeated sequences were analyzed by ROUSFinder2.0.py with default parameters [25], which using BLASTN with a word size of 50, ungapped, no masking, reward +1, penalty −20, and e-value 1000. The plastid derived sequences in mitogenome were performed by blast mitogenome against plastome with e-value cut-off of 1e-6 and a word size of seven.

### 3.3. RNA Isolation, Library Construction and Transcriptome Sequencing

We use single-molecule long-read isoform sequencing (Iso-seq), Illumina RNA sequencing (RNA-seq), and strand-specific RNA-seq (ssRNA-seq) to assess the transcript diversity due to posttranscriptional modifications. For Iso-seq, total RNA from five tissues (stamen, ovary, juvenile leaf, fibrous root, rhizome) of the newly sprouted individual (less than one year) were extracted separately using RNAprep Pure Plant Plus Kit (DP411) (Tiangen, Beijing, China). RNA purity, concentration, and integrity were assessed by 1% agarose gel electrophoresis (200 V, 15 min, D2000 DNA marker), Nanodrop, and Agilent 2100 Bioanalyzer, respectively. cDNA was synthesized using the SMARTer^®^ PCR cDNA Synthesis Kit (Clontech, Palo Alto, CA, USA) and then amplified with optimized PCR cycles by KAPA HiFi^TM^ PCR Kits (KAPA Biosystems, Wilmington, MA, USA). Equimolar fractions of cDNA products from each tissue were pooled and then eight Iso-seq libraries with insert size of 0.5~6 kb were created using the SMRTbell template prep kit 1.0. Un-ligated products were removed by exonuclease and then purified by AMPure PB beads. A single SMRT cell was used for each library and then sequenced on the PacBio Sequel system.

For RNA-seq, total RNA from another six tissues (petiole, fibrous root, mature leaf, sepal, petal, pedicel) of the same individual used in Iso-seq mentioned above were also extracted using Tiangen DP411 Kit, following by the assessment procedures as mentioned above. mRNA was enriched using Oligo (dT)-attached magnetic beads. After fragmentation, mRNA was reverse-transcribed into double-strand cDNA. Six cDNA libraries (one library per tissue) with insert size of about 240 bp were constructed using NEBNext^®^ Ultra^TM^ RNA Library Prep Kit (NEB, Beverly, MA, USA), and then sequenced on Illumina Hiseq X ten platform with 2 × 150 bp PE mode. Each library generated about 6 Gb data.

For strand-specific RNA-seq (ssRNA-seq), libraries without poly-A mRNA enrichment were additionally constructed, using RNA extracted from juvenile leaf, mature leaf and stamen as mentioned above. Ribosomal RNA was removed by Epicentre Ribo-zero^TM^ rRNA Removal Kit (Epicentre, Madison, WI, USA). Three strand-specific libraries using rRNA-depleted RNA were generated by NEBNext^®^ Ultra^TM^ Directional RNA Library Prep Kit (NEB, Beverly, MA, USA) following manufacturer’s recommendations. Each library generated about 6 Gb (150 bp PE) data after sequenced by Illumina Hiseq X ten platform.

### 3.4. Iso-seq and ssRNA-seq Data Processing and Mapping

Raw reads produced by Iso-seq were processed by Iso-Seq v2 in SMRTLINK 5.0.1 [57]. Subreads with the length < 50 nt were removed and reads of inserts (ROIs) were generated (minPasses = 1, minPredictedAccuracy = 0.8). FLNC (full length, non-chimeric) reads and NFL (non-full length) reads were classified by checking the signals of 5′- and 3′- primers, as well as the poly-A tail in ROIs. Iterative clustering and error correction (ICE) algorithm was used to cluster the FLNC reads into consensus sequences, which were polished using the NFL reads by Arrow algorithm. LoRDEC was employed to correct consensus sequences with all the Illumina RNA-seq reads [64]. All the corrected full-length transcripts (hereafter referred to as “Iso-seq transcripts”) were used for further analysis.

Iso-seq transcripts were mapped to the assembled *N.* ‘Joey Tomocik’ organelle genomes and genes/CDSs by GMAP (version 2020-10-14) [65], ‘gmap_build’ with flag “--circular”, and ‘gmap’ with parameters “-f samse -n 0 --nofails” as suggested in https://github.com/Magdoll/cDNA_Cupcake/wiki/Best-practice-for-aligning-Iso-Seq-to-reference-genome:-minimap2,-deSALT,-GMAP,-STAR,-BLAT#refgmap (accessed date: 29 July 2019). Mapped reads were filtered using ‘samtools view’ with the parameter “-bhF 4”. Only Iso-seq transcripts that could be properly aligned to genes and their flanking sequences were manually selected with the help of Geneious R9.1.8 software suite (Geneious, Auckland, New Zealand).

All ssRNA-seq raw data were filtered to remove adapters and low-quality reads by using fastp [66] with default parameters. We then assembled ssRNA-seq clean reads from three libraries using Trinity [67] with parameters “--SS_lib_type RF”. All three assembled transcripts (hereafter referred to as “Trinity transcripts”) were concatenated, and then mapped to the assembled *N.* ‘Joey Tomocik’ organellar genomes by GMAP with the same parameters mentioned above.

All splicing boundaries of intron-containing genes were manually compared to Iso-seq/Trinity transcripts GMAP mapping results. Necessary modifications were collected to refine the organelle genome annotations.

### 3.5. Prediction of Polycistronic Transcript Unit (PTU)

Based on the Iso-seq transcripts mapping results, those Iso-seq transcripts that mapped to more than one gene (or exon of intron-containing gene) with the same direction were kept as a potential polycistronic transcript. The adjacent and overlapped cistronic transcripts were combined into a full PTU. All PTUs identified by Iso-seq transcripts were compared to Trinity transcripts alignment. Overlapped short PTUs were combined into one PTU. The chloroplast genes clustered in the same transcription direction and occurred in a full PTU were postulated as an operon.

### 3.6. Identification and Structure Prediction of trans-Spliced Group II Introns

Intron types of organelle genomes were predicted by using RNAweasel [68]. Genes containing *trans*-splicing introns and their protein-coding sequences (CDS) were extracted. Iso-seq transcripts were mapped to these extracted sequences by GMAP as mentioned above. Iso-seq transcripts spanned any two gene segments were rendered as direct evidence of *trans*-splicing, and those spanned any two exons within one gene segment were considered as *cis*-splicing. The identified splicing events were further confirmed by Trinity transcripts alignment. The typically bipartite structure of all identified *trans*-splicing introns in organelle genomes was predicted according to the conserved secondary structure of six domains in group II intron of plants [2].

### 3.7. The Identification of RNA Editing Sites

Clean ssRNA-seq reads were aligned to chloroplast and mitochondrial genome using splice-aware aligner, HISAT2 version 2.2.1 [69] with flag “--rna-strandness RF”. Unique-mapped read pairs were kept by samtools (Version 1.10-96-gcc4e1a6) with parameter “−f 0×2” [70]. The potential editing sites were extracted by using SNP-calling in bcftools version 1.12 [70], ‘bcftools mpileup’ with stringent parameters “-a FORMAT/AD, FORMAT/DP, INFO/AD -I -d 10000 -B -q 20 -O v” and ‘bcftools call’ with parameters “-O v -m -V indels”. The extracted SNPs were then processed using REDO v1.0 [71] with parameters “-d 30 -c 5” to confirm the editing sites in CDS/rRNA/tRNA region.

To get the editing sites in intron and intergenic regions, we call all SNPs by ‘bcftools call’ with same parameters mentioned above but adding “-v” flag. Then ‘bcftools filter’ was executed with parameters “-i ‘FORMAT/DP>=20 && FORMAT/AD>=5 && QUAL>=30’”. To get RNA editing efficiency (VAF, variant allele frequency) of all filtered sites, ‘bcftools +fill-tags’ was performed with parameters “-- -t VAF”.

We also identified RNA-editing sites of organelle genomes by full-length transcripts mapping method. RNA-editing sites were recorded when more than two edited Iso-seq transcripts mapped to CDSs/tRNAs, or more than three Iso-seq transcripts mapped to introns/intergenic regions. And for Trinity transcripts, more than five edited transcripts mapped to CDSs/tRNAs or adjacent introns/intergenic regions were counted. For those sites identified by REDO but no Trinity transcripts mapped were removed, and for those sites present in filtered vcf files but not in REDO annotation were kept if they were supported by more the half of the Trinity transcripts. The editing sites identified by Trinity transcripts but not in filtered vcf were manually recovered if they were also located in protein-coding sequences.

To eliminate the false positive editing sites, Illumina DNA-seq reads were mapped to chloroplast and mitochondrial genome using bwa mem [72] with default parameters. Genomic SNP-calling was done by ‘bcftools mpileup’ with parameters “-I -d 10000 -C 50 -E -Q 25 -q 20 -O v” and ‘bcftools call’ with parameters “-O v -m -v -V indels ”. Any RNA-editing sites found in genomic SNPs were removed.

PREPACT3 [73] webserver was also used to predict potential RNA editing events and those algae, moss, and liverwort species were excluded from the reference database. Thresholds were set to predictions from minimally 3 and at least 70% of the references in the commons output.

## 4. Conclusions

By utilizing genomic PacBio long reads and Illumina short reads, we performed deep analysis of plastome and mitogenome of *Nymphaea* ‘Joey Tomocik’. By accurately mapping Iso-seq reads to organelle genomes, annotations by in-silico approach were refined, and multiple partially or fully intron-spliced intermediates were observed, which implied that both *cis*- and *trans*-splicing introns were spliced randomly. We also identified the RNA-editing sites in organelle genomes of *Nymphaea* using strand-specific RNA-seq data by direct SNP-calling and then rechecked by transcript-mapping method. By comparing the characteristics of RNA-editing in both organelles, we inferred that a common evolutionary origin and editing machinery may share by plastid and mitochondria in *Nymphaea*. We further proposed that RNA-editing sites in intron and exon region may splice synchronously, except for some exonic sites which were obstructed by un-spliced intron.

## Figures and Tables

**Table 1:** Comparative statistics of organelle genomes of basal angiosperms.

	Chloroplast Genome					Mitochondrial Genome				
	Atri	NJoey	Ncol	Ssph	Ltul	Atri	NJoey	Ncol	Ssph	Ltul
Accession number	NC_005086	MW644616	MT107631	NC_037145	NC_008326	KF754799–KF754803	MW644617	NC_037468	NC_042758	NC_021152
Genome size (bp)	162,686	159,968	159,842	146,843	159,886	3,866,039	335,042	617,195	1,101,768	553,721
No. of genes (unique)	132 (114)	132 (114)	132 (114)	125 (113)	131 (113)	63	73 (65)	66 (60)	70 (58)	65 (62)
No. of protein genes (unique)	87 (80)	87 (80)	87 (80)	82 (79)	86 (79)	40 (40)	43 (41)	43 (41)	43 (41)	41 (41)
No. of tRNA genes (unique)	37 (30)	37 (30)	37 (30)	35 (30)	37 (30)	10 (9)	24 (21)	20 (16)	22 (14)	21 (18)
No. of rRNA genes (unique)	8 (4)	8 (4)	8 (4)	8 (4)	8 (4)	3 (3)	6 (3)	3 (3)	5 (3)	3 (3)
Genome GC content	38.30%	39.10%	39.14%	39.60%	39.20%	45.90%	48.70%	45.10%	46.40%	47.70%
*cis*-splicing intron (unique)	24 (20)	24 (20)	24 (20)	24 (20)	24 (20)	19 (19) ^1^	19 (19)	19 (19)	18 (18) ^1^	19 (19)
*trans*-splicing intron	19	1	1	1	1	6	6	6	6	6

Note: Atri, “*Amborella trichopoda*”; Njoey, “*Nymphaea* ‘Joey Tomocik’”; Ncol, “*Nymphaea colorata*”; Ssph, “*Schisandra sphenanthera*”; Ltul, “*Liriodendron tulipifera*”. ^1^ *nad1* exon 1 in KF754803, other four exons in KF754801; NC_042758 *cox2* gene has only two intervals.

**Table 2:** PTUs identified in organelle genomes of *N.* ‘Joey Tomocik’.

PTU	Genes Covered by PTU
PTU1_chloroplast	*trnQ-UUG, rps16, trnK-UUU(matK), psbA, trnH-GUG*
PTU2_chloroplast	*psbK, psbI, trnG-UCC*
PTU3_chloroplast	*trnE-UUC, trnY-GUA, trnD-GUC, psbM, rpoB, rpoC1, rpoC2, rps2, atpI, atpH, atpF, atpA*
PTU4_chloroplast	*trnC-GCA, petN*
PTU5_chloroplast	*trnT-GGU, psbD, psbC, psbZ, trnG-GCC*
PTU6_chloroplast	*rps4, ycf3, psaA, psaB, rps14, trnfM-CAU*
PTU7_chloroplast	*atpB, atpE, trnV-UAC, ndhC, ndhK, ndhJ*
PTU8_chloroplast	*rbcL, accD, psaI, ycf4, cemA, petA*
PTU9_chloroplast	*psbE, psbF, psbL, psbJ*
PTU10_chloroplast	*petL, petG, psaJ, rpl33, rps18*
PTU11_chloroplast	*clpP, rps12*_5′(exon 1), *rpl20*
PTU12_chloroplast	*psbB, psbT, psbH, petB, petD*
PTU13_chloroplast	*trnI-CAU, rpl23, rpl2, rps19, rpl22, rps3, rpl16, rpl14, rps8, infA, rpl36, rps11, rpoA*
PTU14_chloroplast	*ycf2*(partial), *ycf15*
PTU15_chloroplast	*rps12*_3′ (exon 2-exon 3), *rps7, ndhB*
PTU16_chloroplast	*rrn16, trnI-GAU, trnA-UGC, rrn23, rrn4.5, rrn5, trnR-ACG*
PTU17_chloroplast	*ndhF, trnN-GUU*
PTU18_chloroplast	*ycf1*(partial), *rps15, ndhH, ndhA, ndhI, ndhG, ndhE, psaC, ndHD*
PTU1_mitochondria	*rps2, nad1*(partial, exon 2-exon 3)
PTU2_mitochondria	*rps10, cox1*
PTU3_mitochondria	*nad3, rps12, nad5*_5′(exon 1)
PTU4_mitochondria	*rpl2, rps19, rps3*
PTU5_mitochondria	*nad1*(partial, exon 4), *atp6*

**Table 3:** Annotated group II introns in organelle genomes in *N.* ‘Joey Tomocik’.

Group II Introns	Plastome Introns	Iso-seq ^1^	Trinity ^1^	Mitogenome Introns	Iso-seq	Trinity
*trans*-splicing	***rps12*-i1**	■	■	*nad1*-i1	□	■
				***nad1*-i3**	■	■
				***nad1*-i4**	□	■
				***nad2*-i2**	□	■
				***nad5*-i2**	■	■
				***nad5*-i3**	■	■
*cis*-splicing	*rps12*-i2	□	■	*nad1*-i2	■	■
	*ycf3*-i1	□	■	***nad2*-i1**	□	■
	*ycf3*-i2	□	■	***nad2*-i3**	□	■
	*clpP*-i1	■	■	*nad2*-i4	□	■
	*clpP*-i2	■	■	***nad5*-i1**	■	■
	***rps16*-i**	■	■	*nad5*-i4	■	■
	*atpF*-i	□	■	*nad7*-i1	■	■
	*ndhA*-i	□	■	***nad7*-i2**	□	■
	*petB*-i	□	■	*nad7*-i3	■	■
	*petD*-i	□	■	***nad7*-i4**	■	■
	*rpl16*-i	□	■	***nad4*-i1**	■	■
	*rpoC1*-i	□	■	***nad4*-i2**	■	■
	*trnG-UCC*-i	□	□	***nad4*-i3**	■	■
	*trnK-UUU*-i	□	□	*cox2*-i1	■	■
	*trnL-UAA*-i	□	□	***cox2*-i2**	■	■
	*trnV-UAC*-i	□	□	***ccmFC*-i**	□	■
	^2^*ndhB*-i	□	■	*rpl2*-i	■	■
	^2^*trnA-UGC*-i	□	□	***rps3*-i**	■	■
	^2^*trnI-GAU*-i	□	□	***rps10*-i**	□	■
	^2^*rpl2*-i	■	■			

Note: Introns with identified RNA-editing sites are in bold; ^1^ Iso-seq, intron splicing supported by Iso-seq transcripts, Trinity, intron splicing supported by Trinity assemblies of three ssRNA-seq; ■ detected, □, not detected; ^2^ These introns have two copies.

## Data Availability

The chloroplast genome and mitochondrial genome of *Nymphaea* ‘Joey Tomocik’ has been submitted to GenBank under the accession number of MW644616 and MW644617, respectively. The raw data has been deposited in the Short Read Achieve (SRA) database of NCBI (SRR15402840-SRR15402847).

## Supplementary Materials

Supplementary Materials are available online at https://www.mdpi.com/article/10.3390/ijms22189842/s1.
