# Supplementary material for: Organelle Genomes and Transcriptomes of Nymphaea Reveal the Interplay between Intron Splicing and RNA Editing
Source: Int J Mol Sci. 2021 Sep 11;22(18):9842. doi: 10.3390/ijms22189842 (PMC8466565; doi:10.3390/ijms22189842)
Supplement: Supplementary file 1 [file ijms-22-09842-s001.zip › ijms-1359017-supplementary-FinalProof.pdf]

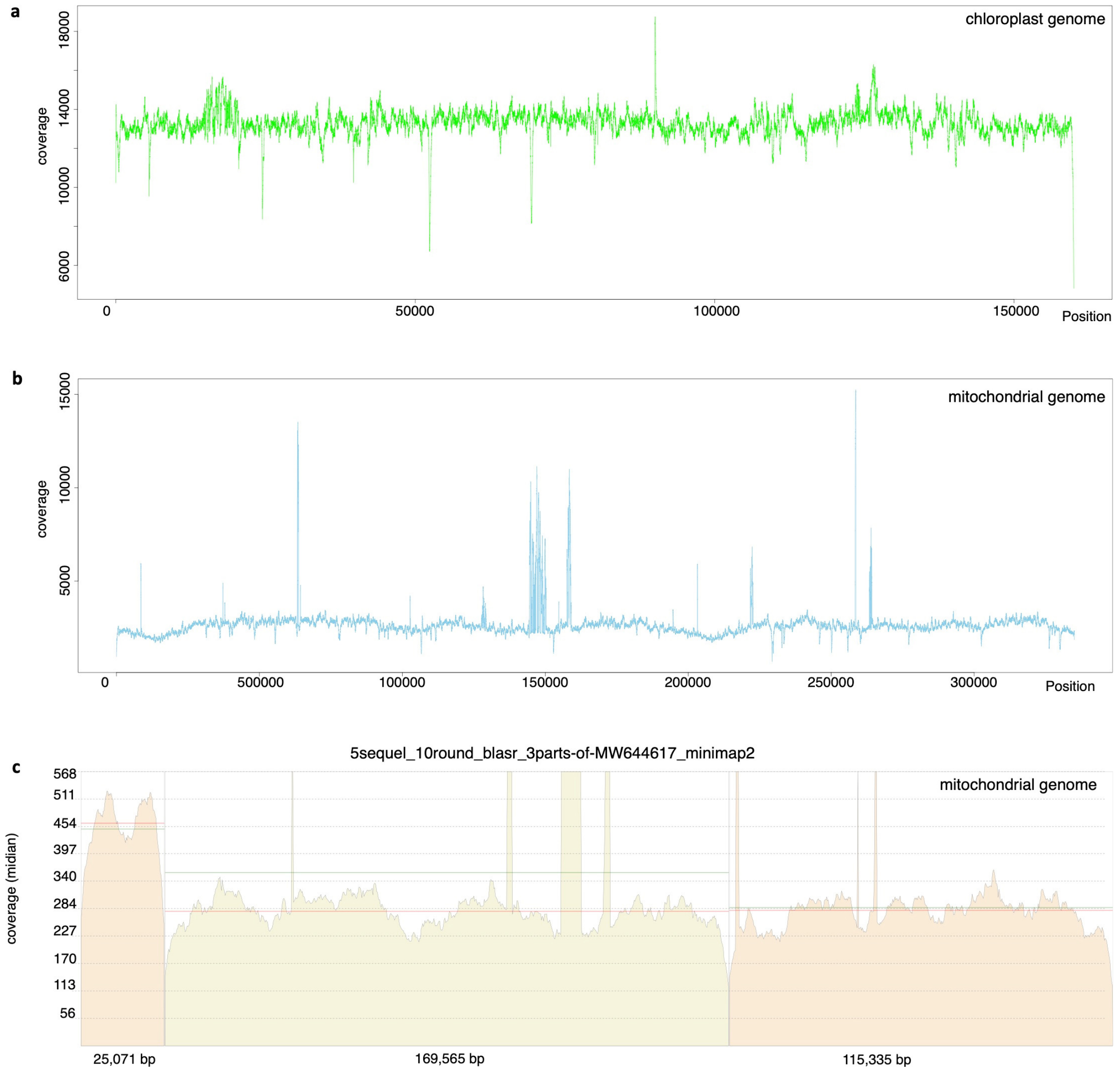

Figure S1. Sequence coverages of plastome (**a**) and mitogenome (**b**) of *Nymphaea* 'Joey Tomocik' from Illuminia short sequences or recruited PacBio long reads (**c**).

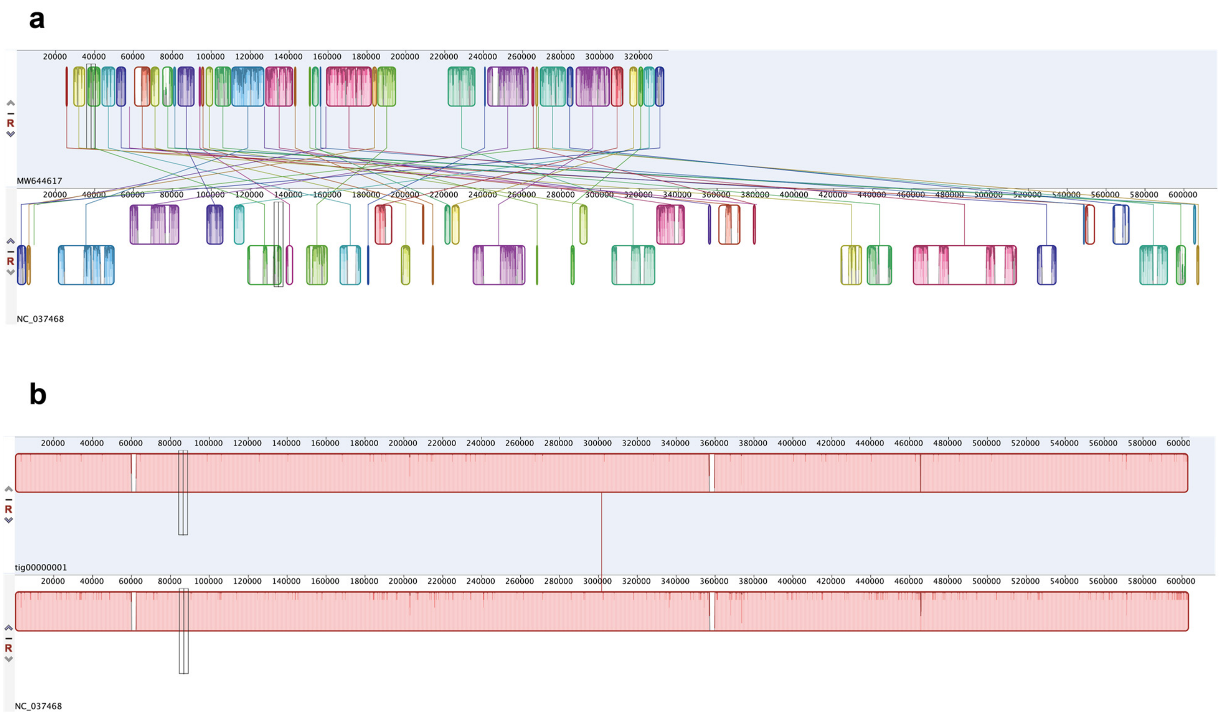

Figure S2. Comparison of two mitogenomes of *Nymphaea*. (**a**) Mauve alignment of *N.* 'Joey Tomocik' and *N. colorata*; (**b**) Mauve alignment of newly assembled *N. colorata* mitogenome in this study and the published one (NC_037468).

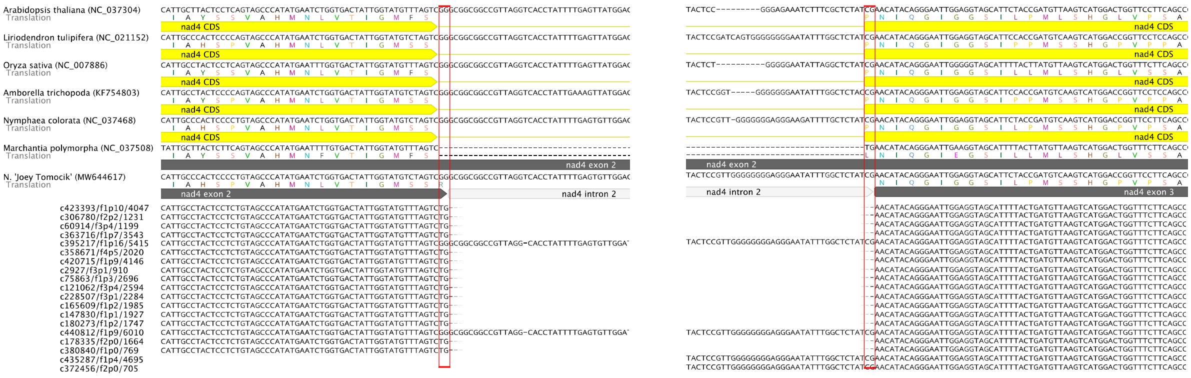

Figure S3. Correction of annotation of *nad4* gene exon 2 and exon 3 using Iso-seq evidence. Red box shows the addition of GG to the end of exon 2 and removal of CG at the beginning of exon 3, resulting amino acid from Pro (CCG) to Arg (CGG).

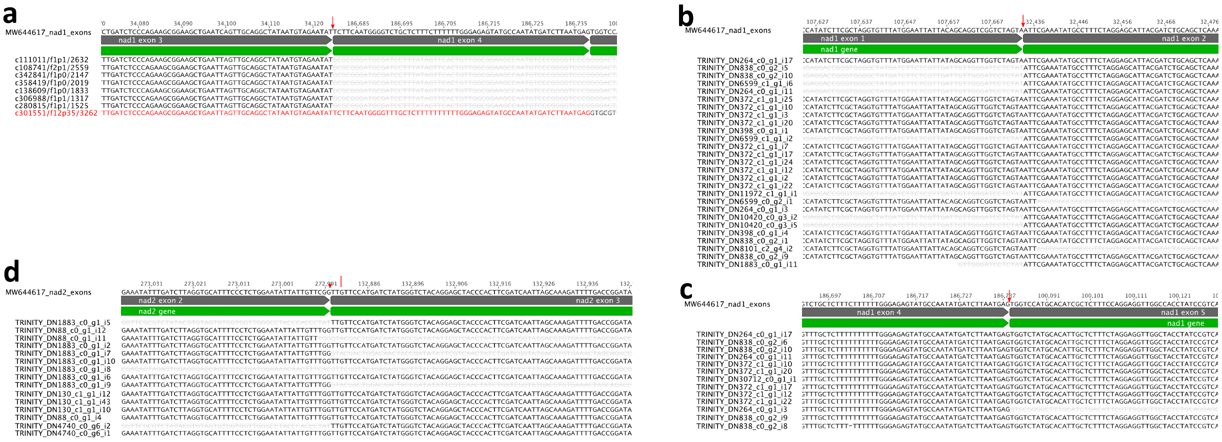

Figure S4. Selected four *trans*-splicing in mitogenome of *N.* 'Joey Tomocik'. (**a**) *nad1*-i3 by Iso-seq transcripts; (**b, c**) *nad1*-i1 and *nad1*-i4 by Trinity transcripts; (**d**) *nad2*-i2 by Trinity transcripts.

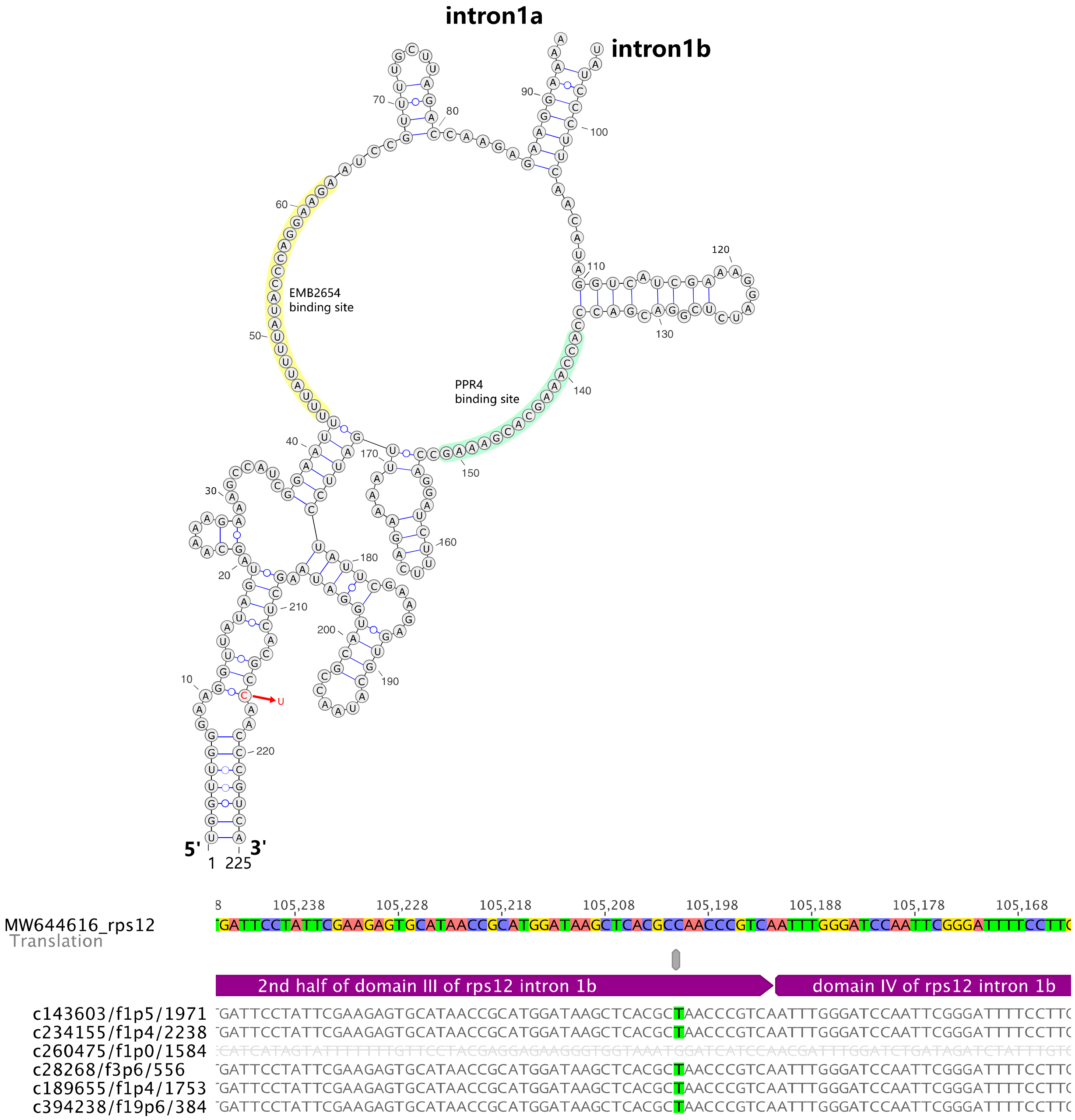

Figure S5. RNA-editing site in *rps12*-i1 domain III, modified from Lee et al. [1].

1. Lee, K.; Park, S. J.; Colas des Francs-Small, C.; Whitby, M.; Small, I.; Kang, H., The coordinated action of PPR4 and EMB2654 on each intron half mediates trans-splicing of rps12 transcripts in plant chloroplasts. The Plant Journal 2019, 100, (6), 1193-1207.

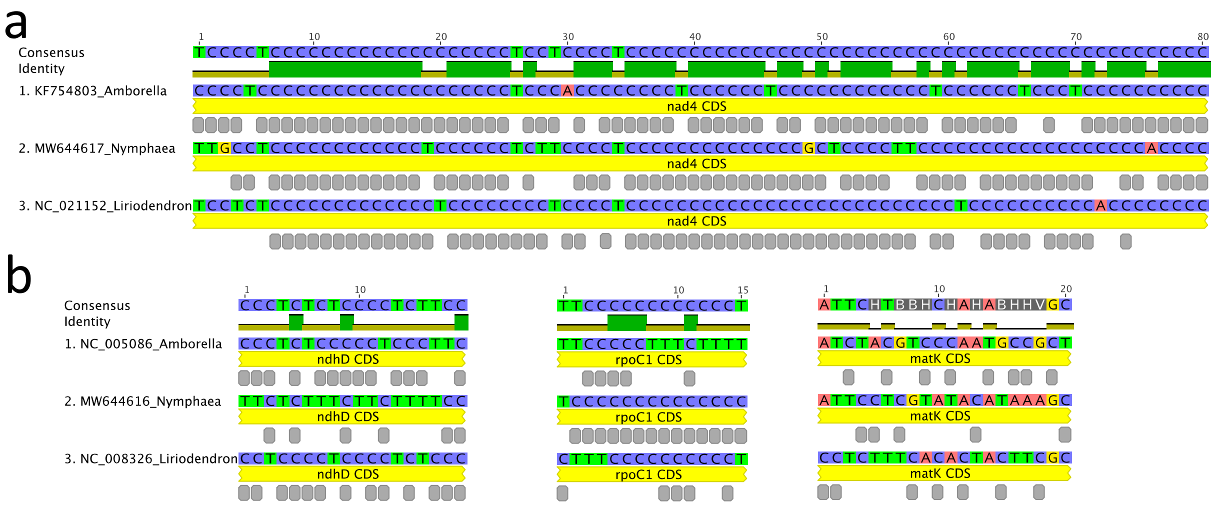

Figure S6. Alignment view of extracted RNA-editing sites in representative genes among three basal angiosperms. RNA editing sites are indicated as grey squares.

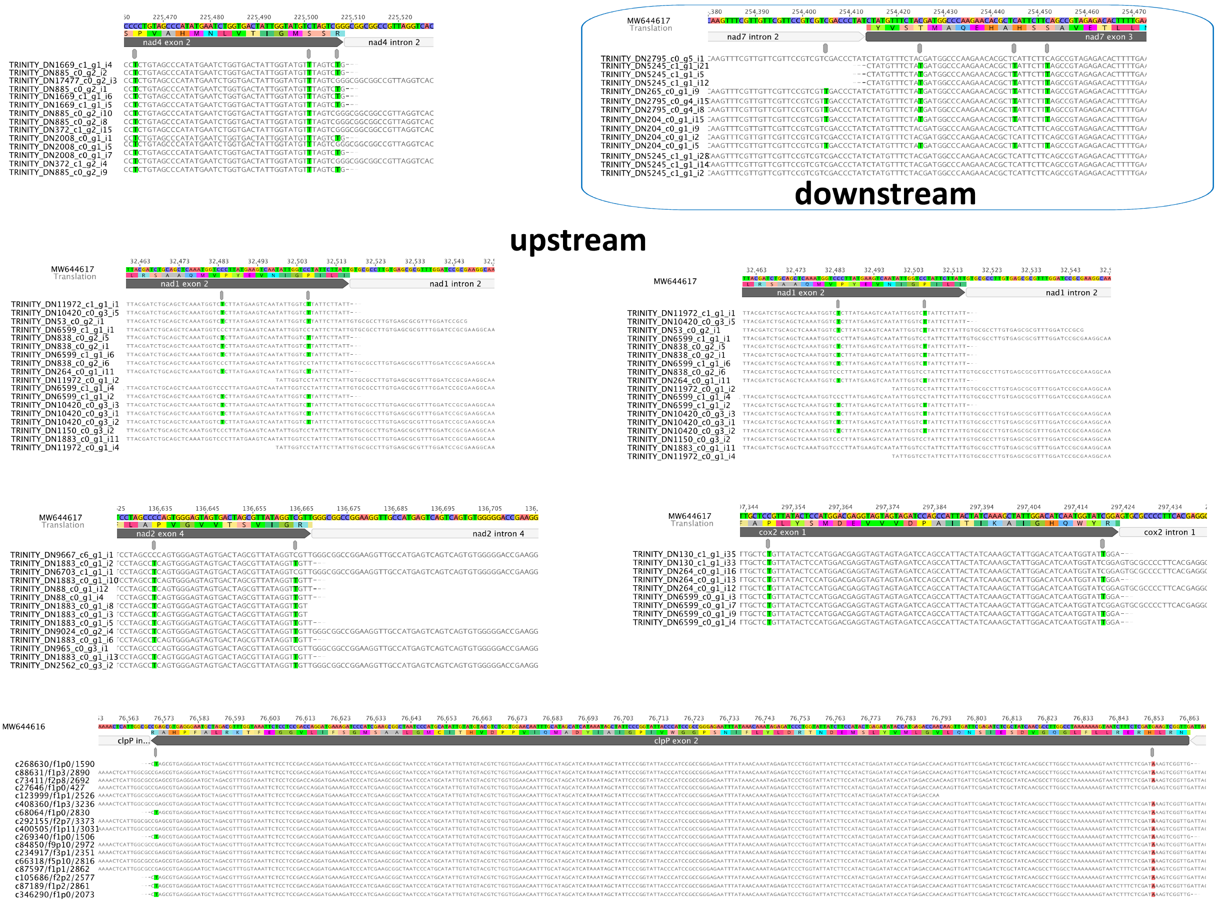

Figure S7. Other exonic RNA-editing sites affected by nearby intron splicing.

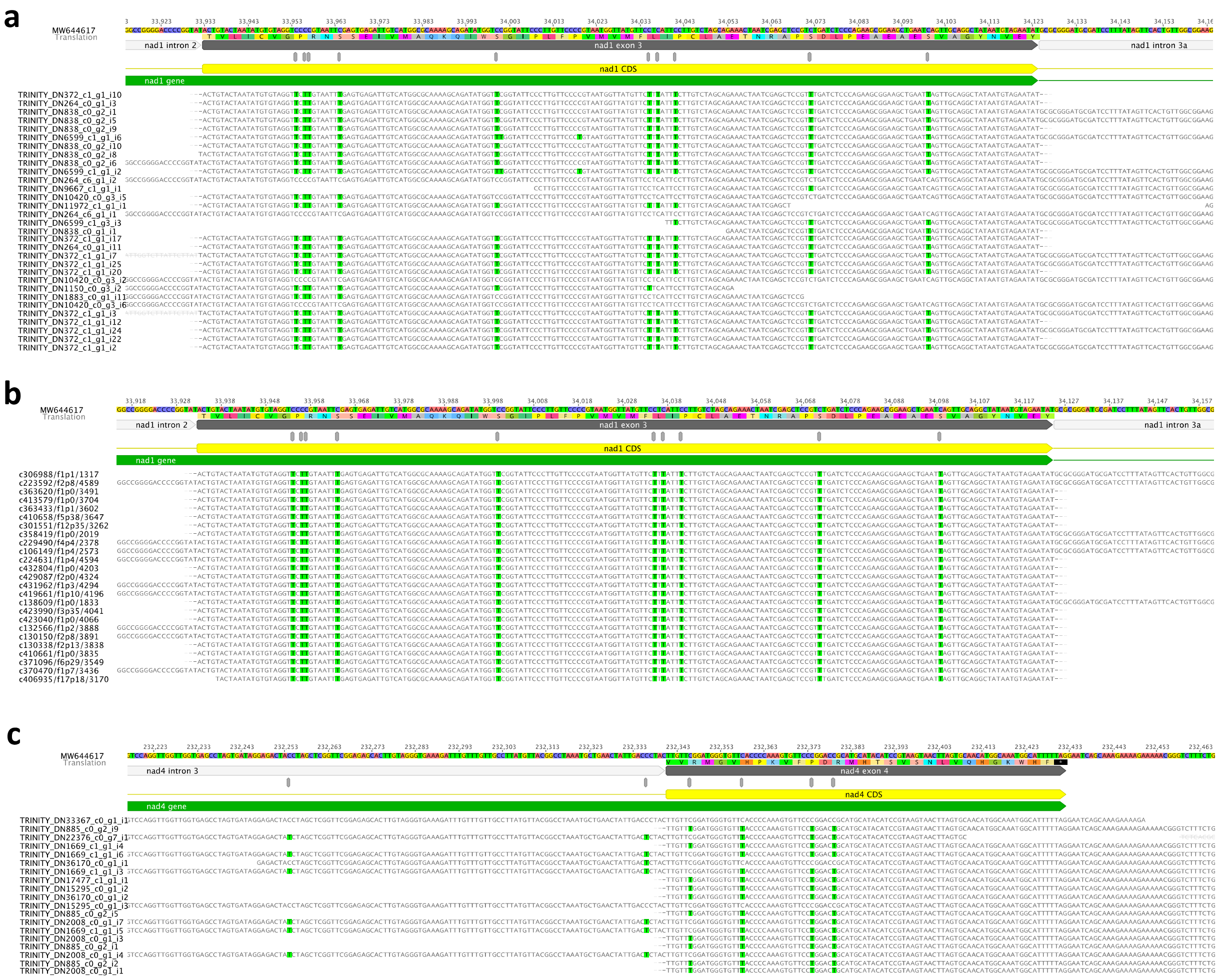

Figure S8. Exonic RNA-editing sites affected by nearby intron by mapping Trinity transcripts or Iso-seq transcripts to reference sequences.

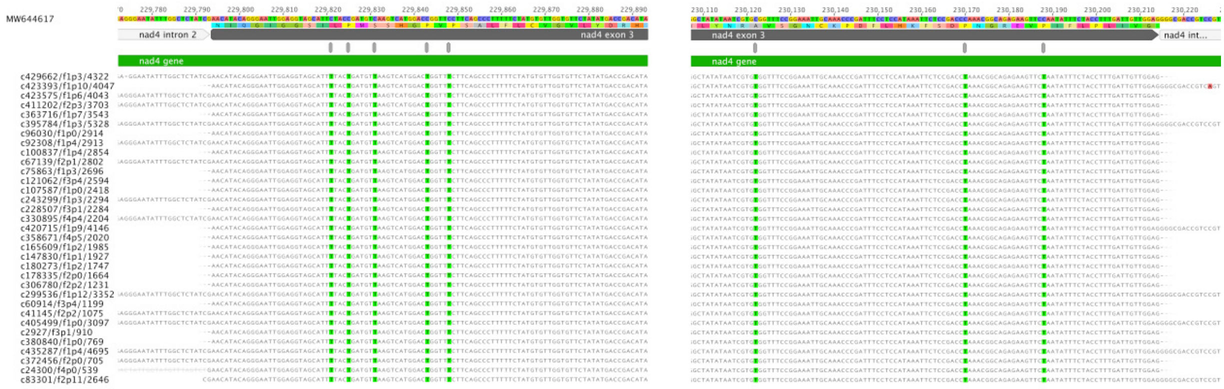

Figure S9. Totally edited sites in exons nearby intron.

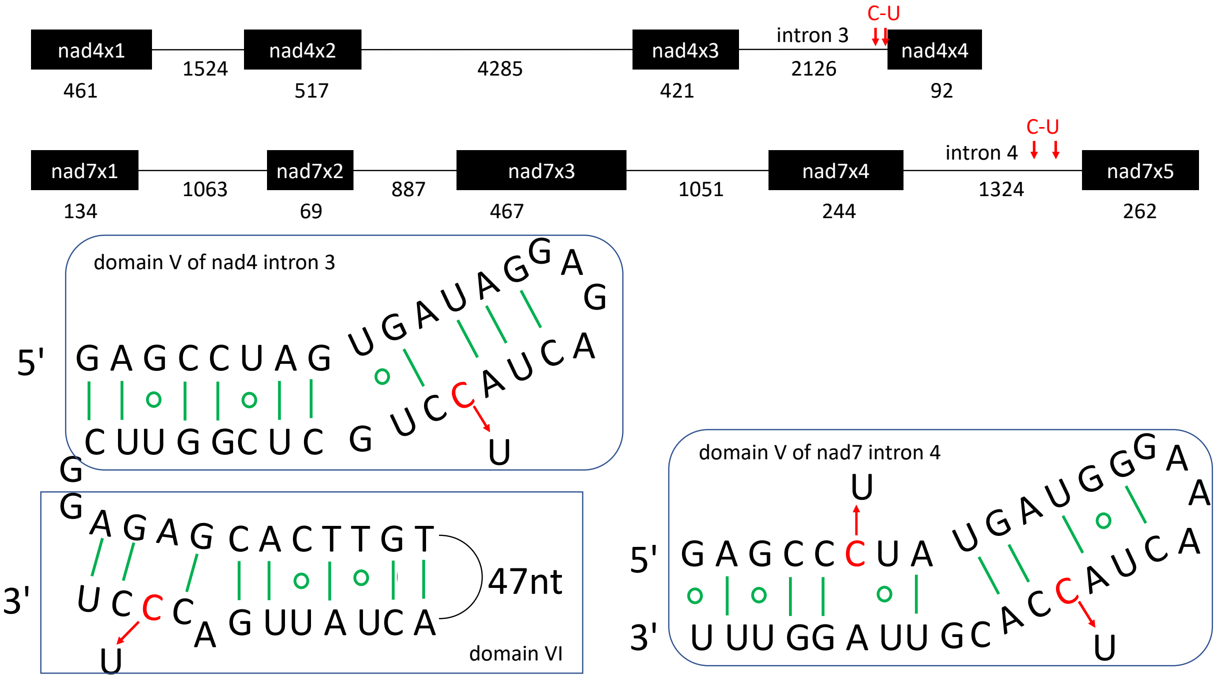

Figure S10. RNA-editing sites in domain V and VI of *nad4*-i3, and domain V of *nad7*-i4.
